# Visual Assessment of Rib Cartilage Mineralization in Thoracic Radiographs as an Indicator of Age in Juvenile Dogs of Various Breeds

**DOI:** 10.1111/vru.70177

**Published:** 2026-04-22

**Authors:** Monika Isabel Hoppe, Carolin Fischer, Mareike‐Kristin Marx, Sebastian Schaub

**Affiliations:** ^1^ Department of Radiology Small Animal Surgery Clinic Justus‐Liebig‐University Gießen Germany

## Abstract

Anecdotally, the onset of mineralization of rib cartilage, as visualized by radiographic studies, is assumed to occur at 3 months of age. Determining radiographically the exact day when rib cartilage mineralization begins in juvenile dogs could aid in age estimation of young dogs with unknown histories. This retrospective study aimed to establish the radiographic onset and progression of rib cartilage mineralization. A number of 1310 dogs across 132 different breeds were included, and 2463 laterolateral or orthogonal radiographs of newborn to 400‐day‐old dogs were evaluated. Rib cartilage mineralization was divided into three grades (I: no radiographic evidence of mineralization; II: partial mineralization [speckled or fine linear mineralization]; and III: complete rib cartilage mineralization). Dogs were divided into five breed groups (toy, small, medium, large, and giant) depending on the estimated final body weight. The earliest radiographically visible rib cartilage mineralization in all dogs was 71 days for Grade II and 132 days for Grade III. Between 132 and 168 days, all three grades of rib cartilage mineralization were present. After 223 days, only complete rib cartilage mineralization was observed. Statistical analysis showed that small‐breed dogs had significantly earlier onset and complete rib cartilage mineralization than medium, large, and giant breeds (*p* < 0.02). Chondrodystrophic dogs of the toy, small, and medium‐breed groups showed significantly earlier onset of costal cartilage mineralization than non‐chondrodystrophic dogs of the same breed group (*p* < 0.001). The predominance of chondrodystrophic breeds among small dogs may explain their earlier rib cartilage mineralization.

## 1 Introduction

Previous studies have confirmed progressive rib cartilage mineralization in different species [1, 2, 3, 4]. In rats, mineralization is visible by the age of 20 days, whereas in humans, the onset of rib cartilage mineralization is described as occurring in late puberty and progressing individually [5, 6]. Costal cartilage mineralization in humans can aid in age estimation. Furthermore, gender‐dependent patterns in costal cartilage have been found in humans [7, 8, 9, 10]. In dogs, costal cartilage is clearly visible on thoracic radiographs: 13 pairs of ribs, nine sternal or true ribs, and four asternal or false ribs are connected to the sternum via costal cartilage and the costal arch [11]. Complete mineralization of the rib cartilage has been reported by the age of 6 months in a population of beagle dogs without any further detailed description [4]. In veterinary medicine, different approaches to age determination are used; evaluating the eruption time of deciduous and permanent teeth is a standard, noninvasive method [12, 13, 14]. Radiographic evaluation of the pulp‐to‐tooth‐width ratio of the maxillary canine teeth is an alternative that has shown promising results in young adult dogs. However, general anesthesia is required to obtain dental radiographs [15]. Additionally, in fully grown animals, an annual thickening of the cemental layer of the tooth (cementum annuli) is reported, which can be counted microscopically and used for age determination. This method is described as reliable in several species; however, tooth extraction and an experienced observer are required [16, 17, 18]. Additionally, the radiographic evaluation of the ossification centers in the long bones of juvenile dogs has also been proposed [14]. Radiographically detectable long bone ossification times have been well defined in veterinary literature [19] and recently updated: The appearance of the ossification center of the paws, stifle, and elbow joint, or the closure of the physis of the humeral condyle, anconeal process, radial carpal bone, and distal femoral epiphysis are most reliable for assessing age [20]. Breed‐specific growth rates and ossification times have been described, with small‐breed dogs being fully grown from approximately 9 months of age, whereas giant‐breed dogs continue growing up to 15 months of age [21, 22].

Forensic and clinical age determination in young dogs is becoming increasingly important in daily veterinary practice due to the rise in illegal dog trafficking, often using forged identification documents. Simultaneously, the adoption rate of dogs with unknown histories from shelters in foreign countries is increasing [23, 24]. Given the age‐related differences in rib cartilage mineralization across species and the wide range of ossification times in dogs, a more precise and easily applicable method for age determination is desirable. Therefore, in cases with indications for thoracic radiographs, the evaluation of rib cartilage mineralization may offer a new prospect for determining age in young dogs without the need for additional studies [25, 26, 27]. To the author's knowledge, no detailed analysis of exact rib cartilage mineralization times in days in different dog breeds has been described. Hence, this study aimed to determine the mineralization times of rib cartilages of juvenile dogs in days and to identify breed‐ and sex‐specific differences.

## 2 Materials and Methods

### 2.1 Case Selection and Description

This study was analytical and retrospective. Due to its retrospective nature, approval by an ethical commission was not required. All thoracic radiographs were obtained from the Small Animal Clinic for Internal Medicine and Surgery hospital database at Justus‐Liebig University in Giessen between December 2005 and January 2024. Radiographs were acquired with the following systems: PCR Eleva, Philips Medical Systems Computed, Best, the Netherlands; Console Advance DR‐ID 300 CL, Fujifilm Medical Systems, Stamford, USA; and Fluorospot Compact FD MultixF‐10500, Siemens Healthcare, Erlangen, Germany. All radiographic systems used were digital radiographic systems. Due to the retrospective nature of the study, no clear protocol was used for the thoracic radiographs; however, for inclusion, the radiographs had to meet the following criteria: laterolateral thoracic radiographs or survey radiographs with collimation of at least 2/3 of the thoracic anatomical structures. Only a slight rotation of the images was considered appropriate. Additional radiographs, like ventrodorsal and dorsoventral projections of the thorax, were not mandatory but were evaluated when available. Included radiographs had to show adequate resolution for visibility of fine mineral structures within the rib cartilages. Radiographic images with inadequate contrast, for example, overexposure leading to decreased visibility of the fine mineralization changes of the rib cartilages, or underexposure leading to a decreased signal‐to‐noise ratio or quantum mottle, were excluded. Artifacts, like motion artifact, also lead to exclusion of the radiograph. The radiographic report either stated “normal radiographic findings of the thorax” or only minor findings, not affecting the assessment of the rib cartilages, for example, a slight interstitial lung pattern. Exclusion was based on clinical history or radiological findings of severe diseases, such as bronchopneumonia or thoracic effusion, which led to effacement of the rib cartilage and artifacts due to overlying ECG cables, feeding tubes, or contrast media. Furthermore, dogs with generalized osteopenia were also excluded. Breeds with a wide variety of appearance without exact specification (e.g., “poodle” without specification of exact estimated final size, e.g., “miniature poodle”) were excluded. As it is described that castration can affect the physeal closure times of young dogs, castrated dogs were excluded to avoid potential confounding in the statistical analysis [28, 29]. Cases were selected between 0 and 400 days of age (365 days + 10%) to obtain definite results about rib cartilage mineralization up to 1 year.

### 2.2 Data Recording and Analysis

#### 2.2.1 Patient and Medical Record Analyses

Before radiographic evaluation, the following patient data were retrieved from the hospital's patient database: patient number, date of birth, date of radiographic examination, radiographic positioning, age in days at the date of the radiographic examination, sex, breed, and weight.

#### 2.2.2 Radiographic Analyses

**FIGURE 1:**
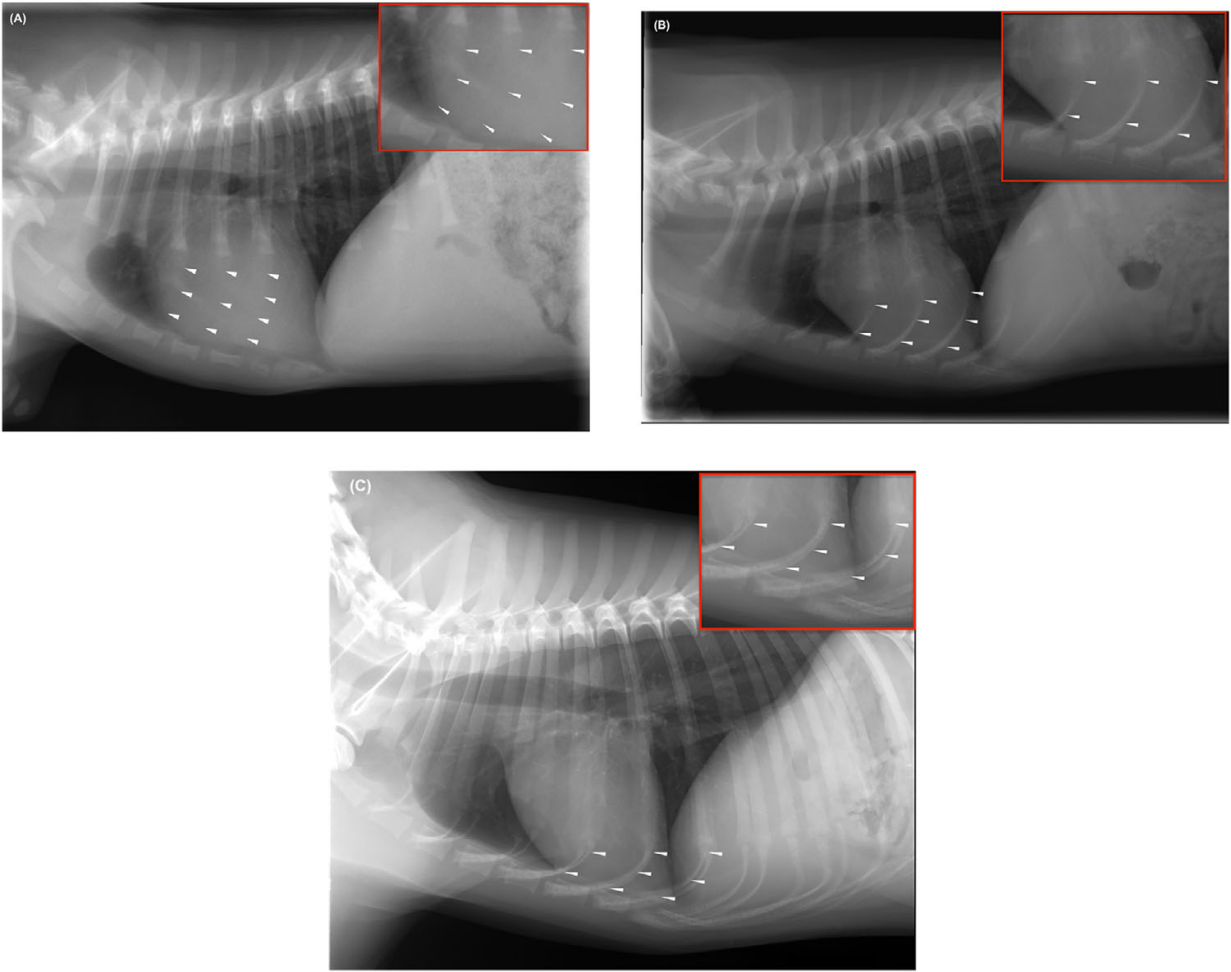
Right lateral thoracic radiographs of juvenile dogs. (A) Grade I mineralization: Rib cartilages are not mineralized: Ill‐defined, faint, soft tissue opaque stripe‐like visibility of the costal cartilages. (B) Grade II mineralization: Partial rib cartilage mineralization with fine lines or speckled mineralization is visible. The mineralized parts of the rib cartilages are not in contact with the ribs. (C) Grade III mineralization: Complete rib cartilage mineralization with well‐visible, well‐delineated, and homogenous mineralization of rib cartilages, reaching dorsally to the ends of the ribs.

A grading scheme was proposed to classify the degree of mineralization of the rib cartilage. Grade I has no apparent mineralization; only faint, ill‐defined, soft tissue opaque cartilage is visible. Grade II shows the onset of rib cartilage mineralization, which often appears as speckled or as a fine line at the mid‐level of the cartilage, but is not in contact with the ribs. Grade III shows complete, homogeneous mineralization reaching dorsally to the end of the ribs. Examples are provided in Figure 1.

A first‐year resident (M.I.H.) of the European College of Veterinary Diagnostic Imaging (ECVDI) selected the radiographs and evaluated them for the degree of mineralization (I, II, or III) of the costal cartilage under the supervision of two board‐eligible specialists in veterinary diagnostic imaging (M.K.M./C.F.). Debatable cases were later discussed in a group with a board‐certified specialist of the ECVDI (S.S.).

All images were viewed at an imaging workstation using DICOM viewing software (iMac Retina 5K, 2015, 27‐inch, Apple, California; Horos 3.3.6). The observers could adjust post‐processing parameters, such as the window width, level, and magnification. The observers were blinded to the patient's age due to prior randomization of cases with a random number generated using Excel (Microsoft, Excel 16.87, Redmond, Washington, USA).

### 2.3 Statistical Methods

Statistical analysis was performed by a first‐year ECVDI resident (M.I.H.) using commercially available software (IBM, SPSS Statistics, Version 28.01, Armonk, New York, USA). Furthermore, MATLAB (Mathworks, Version 2022b, Natick, Massachusetts, USA) was utilized to visualize logistic models. Grading of the costal cartilage mineralization, radiographic positioning, age, weight, sex, and breed were analyzed. In this article, 1 month is assumed to be 30 days. The data were tested for normality with the Kolmogorov–Smirnov test and *Q*–*Q* plots. The level of significance was set at *p* ≤ 0.05.

First, the costal cartilage mineralization grades, radiographic positioning, age, weight, sex, and breed of the entire group were analyzed descriptively. The Pearson correlation coefficient *r* for weight versus age was determined (0.1–0.3, weak correlation; 0.3–0.5, medium correlation; >0.5, strong correlation). As a second step, dogs expected to have similar properties were clustered into breed groups according to the anticipated weight of fully grown individuals (toy < 5 kg, small 5–10 kg, medium 10–25 kg, large 25–40 kg, and giant > 40 kg). Because mixed breeds are a combination of unknown dog breeds and display wide diversity in final size, they were not included in further statistical analysis. Chondrodystrophic dogs were included in the breed groups according to their expected final weight.

Mean ages for the onset of mineralization and complete mineralization in different groups were analyzed using ANOVA to compare several means. The ANOVA effect size was measured with Cohen's *f* (*f* = 0.1, weak; *f* = 0.25, medium; and *f* = 0.4, strong). Additionally, the results were verified with the nonparametric Kruskal–Wallis test and the post hoc test with a Bonferroni correction. The onset time of mineralization and complete mineralization, dependent on sex and chondrodystrophy, was evaluated with a *t*‐test and a Mann–Whitney *U* test. Effect sizes for nonparametric tests, the *t*‐test, and the correlation tests were classified using Pearson's *r* (*r* = 0.1 weak, *r* = 0.3 medium, and *r* > 0.5 strong effect). To obtain predictive models for the age dependence of rib cartilage mineralization in young dogs, logistic regression analysis was performed using the state “complete rib cartilage mineralization” (member of the Grade‐III‐group) or not (member of Grade‐I‐ or Grade‐II‐group) as an age‐dependent binary indicator variable. The models give probabilities to find a completely mineralized dog with a certain age in different breed groups, using age, sex, weight, and chondrodystrophy as predictive parameters. The validity of the whole model and the coefficients for the aforementioned predictors were tested with the chi‐squared or the Wald test, respectively.

## 3 Results

### 3.1 Descriptive Statistics of Radiographic Observations, Including All Dogs

Within the hospital database, 2199 dogs were found, of which 1310 met the inclusion criteria. In all, 132 different breeds were included, averaging eight dogs per breed. The most common breeds were mixed breed (251, 19.2%), Labrador Retriever (89, 8.4%), German Shepherd (54, 5.1%), French Bulldog (79, 7.5%), Chihuahua (62, 5.9%), Australian Shepherd (36, 3.4%), Dachshund (35, 3.3%), and Golden Retriever (33, 3.1%). A detailed list is provided in Supporting Information Table A1.

At the time of the examination, the youngest dog was 4 days old, and the oldest was 395 days old. The average age was 143.64 days (±72.18 days). The most common radiographic views were right lateral and dorsoventral (815, 61.99%), right lateral and ventrodorsal (289, 22.69%), and right lateral only (160, 12.23%) (Supporting Information Table A2). There were 662 (50.5%) females and 648 (49.5%) males (Supporting Information Table A3).

Of 1310 dogs, 251 (19.16%) were mixed breed dogs, and the remaining 1059 dogs were sorted into their breed groups: toy breeds (133, 10.15%), small breeds (185, 14.12%), medium breeds (306, 23.35%), large breeds (367, 28.02%), and giant breeds (68, 5.2%). A number of 328 (30.97%) of 1059 dogs assigned to their breed groups were chondrodystrophic. Chondrodystrophy occurred only in small, toy, and medium breeds (Supporting Information Table A4). The Kolmogorov–Smirnov test rejected the hypothesis of a normal distribution of age data (*p* < 0.05) (Supporting Information Tables A14 and A15).

Within the whole data set, including mixed breeds, a total of 488 dogs (37.25%) were categorized as Grade I rib cartilage mineralization, 517 dogs (39.47%) were classified as Grade II, and 305 dogs (23.28%) were designated as Grade III. Including all dogs, the mean age in days with mineralization Grade I was 80.27 ± 29.46 days old; dogs with mineralization Grade II averaged 148.02 ± 31.87 days of age, and dogs with mineralization Grade III had a mean age of 236.90 ± 64.86 days (Tables 1 and 2).

**TABLE 1:** Overview of the number of individuals per breed group and average age for the onset and complete rib cartilage mineralization.

Breed groups		Age in days at mineralization Grade I	Age in days at mineralization Grade II	Age in days at mineralization Grade III
Small	Mean (days) ± SD	80 ± 24	134 ± 34	205 ± 56
	*n*	64	69	52
	Minimum–maximum	24–156	71–223	132–390
Toy	Mean (days) ± SD	81 ± 25	139 ± 32	235 ± 71
	*n*	39	66	28
	Minimum–maximum	26–153	89–217	154–387
Medium	Mean (days) ± SD	82 ± 26	148 ± 29	247 ± 62
	*n*	103	133	70
	Minimum–maximum	28–148	92–213	154–388
Large	Mean (days) ± SD	79 ± 30	157 ± 29	242 ± 66
	*n*	171	119	77
	Minimum–maximum	4–168	91–219	161–391
Giant	Mean (days) ± SD	91 ± 32	165 ± 29	270 ± 66
	*n*	28	25	15
	Minimum–maximum	27–153	122–217	194–391

**TABLE 2:** Overview of the number of individuals per grading group and average age for the onset and complete rib cartilage mineralization without mixed breeds.

Grades	*n* (Number of individuals)	Mean and standard deviation	Median	Minimum	Maximum
I	405	80 ± 29	82	4	168
II	412	148 ± 32	145	71	223
III	242	236 ± 65	208	132	391

Weight is not an independent predictor of mineralization as all dogs grow with age. The Pearson correlation (weight vs. age) is significant, with a strong correlation (*p* < 0.001; *r* = 0.549); hence, weight was discarded from further analysis.

### 3.2 Radiographic Analysis Statistics of Dogs in Specific Breed Groups, Excluding Mixed Breeds

#### 3.2.1 Overall Age and Sex Distribution of Breed Groups

**FIGURE 2:**
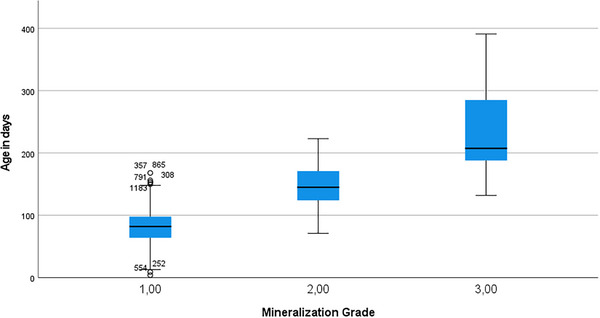
Box plot for grading stages versus age in specific breed groups: The line marks the median age within the grading stage, whereas the whiskers mark the minimum and maximum ages within the group. Grade I shows outliers, labeled with their row number in the data set.

The box plot (Figure 2) shows the age of all pure‐bred dogs in our sample on the day of radiographic examination, sorted into three mineralization grades. In our sample, the youngest dog in Grade II was 71 days old, and the youngest dog in Grade III was 132 days old. The oldest dog with unmineralized rib cartilage (Grade I) was 168 days old. Hence, from 132 to 168 days, radiographs from all three mineralization grades were encountered in this sample (Table 2). At 223 days of age, the mineralization process can be considered complete because no dog from Grade I or II is visible on the graph beyond this day. Grades II and III show overlapping distributions, with both mineralization grades found between 132 and 223 days. Female dogs in Grade II show a significantly earlier mean onset of mineralization (*t*‐test: *t*[410] = 3.433, *p* = 0.001) compared to males, with dates of 142.8 ± 32.1 days versus 153.4 ± 30.5 days. A difference of 10.5 days with a weak effect (*r* = 0.167) was noted. There was no significant difference between sexes in Grade III for complete mineralization age (*t*‐test: *t*[240] = 0.627, *p* = 0.531) (Supporting Information Tables A12 and A13).

#### 3.2.2 Grade II and III Mineralization Ages in Different Dog Breed Groups

**FIGURE 3:**
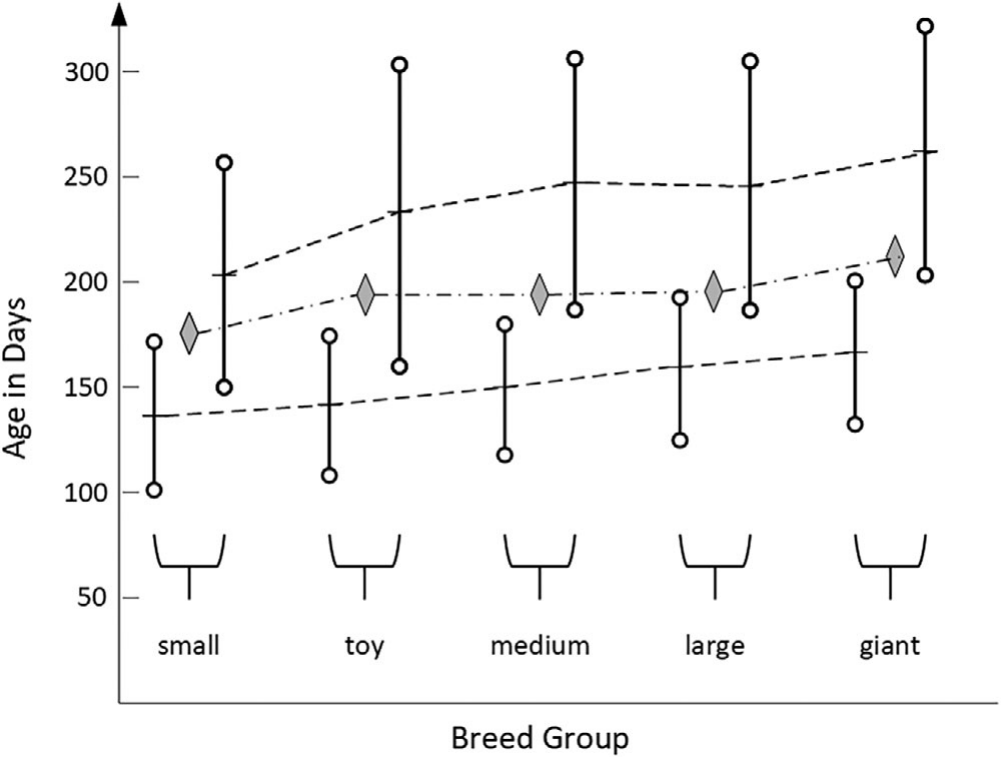
Mean values and standard deviations in Grade II (bottom row) and Grade III (upper row) for all breed groups. Diamonds indicate the *t*0.5 values from logistic regression in each breed group.

The mean age in days for Grade II and III mineralization was compared between the breed groups (Figure 3, Supporting Information Tables A6 and A7). Univariate ANOVA showed significant differences with a medium effect size (*f* = 0.31) in the mean age at the onset of mineralization (*F*[4.407] = 9.843; *p* < 0.001;

$${\eta^{2}}_{p}$$

η^2^_*p*_ = 0.088, *n* = 412). According to post hoc testing with Bonferroni correction, small breeds in our sample have a significantly earlier onset of rib cartilage mineralization (mean age 134 days) compared to medium (+15 days, *p* = 0.002), large (+23 days, *p* < 0.001), and giant breeds (+32 days, *p* < 0.001). There were no other significant differences between pairs of breed groups. A nonparametric Kruskal–Wallis test supports these findings.

When the mean age in days for Grade III mineralization of the rib cartilages was compared between the breed groups, univariate ANOVA showed significant differences with a medium effect size (*f* = 0.276) for complete mineralization (*F*[4.237] = 4.528; *p* < 0.002; η^2^_*p*_ = 0.071, *n* = 242). According to post hoc testing with Bonferroni correction, small breeds have a significantly earlier complete mineralization of the rib cartilages (mean age 205 days) compared to medium (+40 days, *p* = 0.006), large (+35 days, *p* = 0.02), and giant breeds (+63 days, *p* = 0.008). The Kruskal–Wallis test showed a significant difference between the distribution of the ages in days for complete rib cartilage mineralization.

#### 3.2.3 Logistic Regression per Breed Group

**FIGURE 4:**
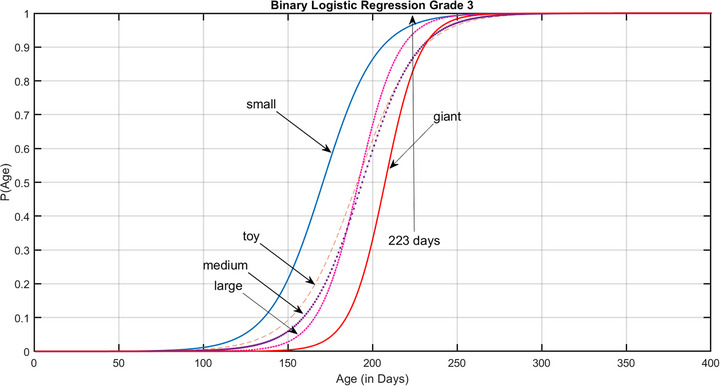
Binary logistic regression model showing the probabilities for reaching Grade III mineralization for different breed groups. The arrow marks Day 223, after which only complete mineralization was noted in the sample population.

Figure 4 shows a binary logistic regression analysis with the outcome completely mineralized rib cartilages (Grade III) or not (Grade I or II), using age as the only predictor. This model correctly classifies 89% of the dogs in the sample. Detailed information on these models is provided in Supporting Information Table A16. Age, weight, and chondrodystrophy as predictors resulted in a significant model as well. However, the percentage of correct classification remained around 89%; hence, more predictors did not improve the model. Sex as an additional predictor was nonsignificant for all breed groups (*p* = 0.155).

*t*0.5 or *t*0.9 is the age in days when the probability of reaching Grade III is 50% or 90%, respectively, with *t*0.5 serving as the expected time for transition to complete rib cartilage mineralization, as indicated in Figure 3 with diamonds. The expected age for the transition to complete mineralization in our sample was earlier in small‐breed dogs than in toy, medium, and large breed dogs (3 weeks earlier) and in giant breeds (5 weeks earlier). Similar results were found for *t*0.9, in which small breeds also showed earlier complete rib cartilage mineralization compared to toy, medium, large, and giant breeds (2 to 3 weeks). The arrow in Figure 4 marks the age of 223 days. After this critical age, only completely mineralized rib cartilages occurred in the dogs of our sample. On this specific date, the probability of reaching Grade III based on the breed groups’ logistic model ranged from 85% to 97%.

#### 3.2.4 Chondrodystrophic Dogs in Grades II and III and Binary Regression Analysis

Earlier onset of rib cartilage mineralization and earlier complete mineralization were observed in chondrodystrophic dogs (138.3 ± 30.9 days for Grade II or 228 ± 68 days for Grade III) in our sample compared to other dogs (152.4 ± 31.2 days for Grade II or 242.1 ± 63.2 days for Grade III) of the same breed group. The *t*‐test for Grade II rib cartilage mineralization showed that the mean difference (*t*[410] = 4.355) of 14.1 days was significant (*p* < 0.001). The effect size *r* = 0.210 indicated a weak effect. The *t*‐test for Grade III rib cartilage mineralization showed that the mean difference (*t*[240] = 1.666) of 14.1 days was not significant (*p* = 0.097). By contrast, the Mann–Whitney *U* test showed significant differences between the complete mineralization times of chondrodystrophic dogs compared to non‐chondrodystrophic dogs (*p* < 0.023; *r* = 0.146) with a weak effect (Supporting Information Tables A9 and A10).

In the binary logistic regression analysis of our sample, *p*(*t*0.5) = 50% was reached in the chondrodystrophic cohort at 180 days, 16 days earlier than in non‐chondrodystrophic dogs at 196 days. Logistic regressions for small‐breed dogs and medium‐breed dogs regarding chondrodystrophy yielded significant models. In toy dogs, the overall model was significant, but the Wald test for coefficients failed (*p* > 0.05; Supporting Information Table A16).

## 4 Discussion

### 4.1 Overall Rib Cartilage Mineralization Time

This study aimed to determine the impact of age on rib cartilage mineralization in juvenile dogs and its potential for age prediction. Age had the most significant impact on rib cartilage mineralization. As juvenile dogs grow and gain weight with age, weight is not an independent predictor of rib mineralization. Mineralization of the rib cartilages in our sample begins at 71 days (Grade II) and 132 days (Grade III). These values are the boundaries in the age scale for the onset of Grade II and III mineralization. They were found for single individuals taken from a large sample size, which should hence be representative. On average, the age of the dogs with Grade I rib cartilage mineralization was 80 days, with Grade II 148 days, and with Grade III 236 days. All dogs older than 223 days (or 7.4 months) exhibited completely mineralized rib cartilage on radiographs. The youngest dog in Grade III is 132 days old, whereas the oldest dog in Grade II is 223 days old. This overlap of 91 days indicates that the transition from partial to complete mineralization can occur over a period of about 3 months.

In comparison with studies investigating the development of single patients with multiple radiographic exposures to find an exact time of ossification center appearance or physeal fusion, a retrospective approach omits serial investigation of the same patients and could give a less precise and broader time frame [30, 31, 32]. Still, to a similar extent, comparable ranges are described for the appearance and fusion of long bone ossification centers. Thrall et al. provide a range of 2–3 weeks for the appearance of ossification centers and about 2–3 months for their fusion [19]. Likewise, ranges up to 10 weeks are described for tooth eruption time [12]. Studies on ossification differences in small and giant breed dogs are still rare. Nevertheless, significant overlap with large breeds is described, making only breed‐specific factors less likely to cause this range [20]. Most likely, the variety found in all three methods for age determination is individual or influenced by confounding variables like diet or individual health state [12, 19, 33].

### 4.2 Breed‐Specific Rib Cartilage Mineralization Time

Second, we aimed to identify breed‐specific differences in costal cartilage mineralization. Small dog breeds showed an earlier onset of rib cartilage mineralization (Grade II) by up to 4 weeks compared to medium, large, and giant breeds. Complete rib cartilage mineralization of small breeds (average age 205 days) was observed 4 weeks before the average, formed by toy, medium, and large breeds (about 241 days), and 8 weeks before giant breeds (269 days). The mentioned values should be viewed as a breed‐group‐specific, reliable average obtained by a comparable large number of individuals in different breed groups. Still, the breed groups combine several breeds with similar expected final body weights but possibly different physical appearances, growth rates, or limb conformation, which could lead to false averages for specific breeds. However, because of the large sample size, the earlier rib cartilage mineralization indicated in small breeds is still a representative finding. Previous studies showing differences in growth rates in different dog breed sizes indicate a more extended period of growth for large and giant breeds [21, 22]. Breed‐specific differences in regulatory networks, such as the insulin‐like growth factor (IGF) at growth plates, have been described as contributing to the different growth rates in various dog breeds [34]. These differences could lead to earlier mineralization of the ribs in small‐dog breeds and secondary mineralization of the costal cartilage.

### 4.3 Logistic Regression Analysis

Logistic regression models were derived to identify when complete mineralization can be expected. The results showed that age with odds >1 is the main factor that influences rib cartilage mineralization, whereas predictors like sex, weight, or chondrodystrophy do not increase model accuracy substantially. Logistic regression analysis supports our results for the different mineralization times of various dog breeds found by ANOVA. Small dogs again show an earlier onset and complete mineralization. When our logistic regression analysis is compared to previous findings, matching time frames can be identified: Fukuda [4] described that most of the costal cartilage in his group of beagles showed calcification after 6 months of age. This time frame matches our study, showing complete mineralization at 247 days (~8.2 months) for medium breeds. However, beagles are medium‐sized, chondrodystrophic dogs [35]. In logistic regression analysis of medium‐sized chondrodystrophic dogs, the 0.5 age in days when the probability of reaching complete mineralization is 50% was set at 188 days (6.2 months), equivalent to Fukuda's findings that from this day on, complete mineralization is probable.

### 4.4 Chondrodystrophy and Rib Cartilage Mineralization

Furthermore, an earlier onset of rib cartilage mineralization in chondrodystrophic dog breeds was noted. When non‐chondrodystrophic dogs and chondrodystrophic dogs were compared with logistic regression analysis in their breed group, small chondrodystrophic dogs showed the earliest complete mineralization with 166 days, compared to 183 days for small non‐chondrodystrophic dogs. However, the small‐breed group consists of 75% chondrodystrophic dogs. As the weighted average of 166 and 183 days results in 171 days, the early complete mineralization may stem from the chondrodystrophic fraction in this breed group. In other breed groups (toy and medium), the non‐chondrodystrophic part of the sample has the majority. This could explain the minor differences in the respective rib cartilage mineralization times in these breed groups. To determine the exact time difference of onset and complete rib cartilage mineralization between chondrodystrophic and non‐chondrodystrophic breeds, further comparative, possible prospective imaging studies would be ideal. However, a representative study would require a large sample size, which is only theoretically feasible. It is known that chondrodystrophic dogs show alterations at the growth plates, leading to altered lengths of the long bones and the vertebral bodies [36, 37]. The distal growth plate of the rib has been found to share the morphology of growth plates of long bones like the radius and ulna, and it is highly active because it is solely responsible for the long growth of the ribs [34, 38]. It could be hypothesized that chondrodystrophic growth plates of the distal ribs show the same alterations as the growth plates of long bones, leading to shorter ribs and an earlier beginning of rib cartilage mineralization, compared with other small dogs. However, statistical tests do not confirm earlier complete mineralization, and chondrodystrophy is not a predictor that improves the models’ accuracy for the expected age of rib cartilage mineralization. Therefore, the influence of chondrodystrophy on rib cartilage mineralization has to be evaluated with further studies.

### 4.5 Sex and Weight

Our study found a sex‐specific difference in rib cartilage mineralization times, with a statistically significant 10‐day earlier onset of mineralization in female dogs. However, the ages for complete costal cartilage mineralization did not differ between the sexes. This may indicate a longer mineralization process in females than in males. This matches the results of previous studies, which did not find a difference in complete costal cartilage mineralization times between female and male dogs [4]. Evidence of earlier onset of mineralization in women has been debated in several publications [1, 9, 39]. Furthermore, sex‐specific differences in costal cartilage mineralization patterns are commonly detected [1, 9]. The authors did not notice any sex‐related patterns in canine rib cartilages subjectively.

### 4.6 Statistical Analysis

Notably, when analyzing data from rib cartilage mineralization of Grade III dogs, the mean value of age increases if dogs older than 400 days are included in the study. Hence, the values given with logistic regression analysis are more reliable, as *t*0.5 is a transition time and is therefore not influenced by the number of older dogs in the sample. A 90% probability of complete mineralization is given to overall breed groups for 226 days with logistic regression analysis, whereas the 223‐day period is the cutoff value for Grade II mineralization obtained from the descriptive statistics of the sample data. This correspondence substantiates the validity of the logistic regression model.

As the comparison of mean values is concerned, the Kolmogorov–Smirnov test rejected the hypothesis of a normal distribution of age data (*p* < 0.05). According to Glass [40], ANOVA is robust against slight deviations from normality as encountered in our sample. Parametric test results were confirmed by their nonparametric counterparts and by comparisons with appropriate binary logistic models to exclude false significances due to deviations of the age distributions from normality.

### 4.7 Limitations

This study has certain limitations, starting with its retrospective design. Although a prospective approach with repeated radiographic image acquisition during skeletal maturation of individual patients would have enriched our data, we relied on radiographs of juvenile patients taken for various clinical reasons. This limits the ability to precisely determine the exact time point of rib cartilage mineralization in an individual. A prospective study with serial radiographs at short, predefined intervals would be necessary to address this, but such a study design raises significant ethical concerns due to multiple radiation exposures of healthy juvenile animals without a direct clinical indication, putting them under unnecessary risk for the development of future detrimental health concerns, such as cancer or immunological consequences described in different species [41, 42, 43, 44]. As in human medicine, veterinarians are bound by radiation safety principles, such as ALARA (as low as reasonably achievable), limiting the feasibility of such a study [45]. Furthermore, a large number of individuals would have been necessary in this prospective approach to achieve a statistically significant result, as rib cartilage mineralization is an individual process. A retrospective study with larger sample sizes, especially with truly random selection due to medical indication of thoracic radiographs, could result in more reliable averages. Additionally, it is worth noting that even though many breeds and individuals were considered, specific groups (e.g., giant breeds) held only a few individuals. The rib cartilage mineralization Grade III group was slightly smaller than Groups I and II. This was considered acceptable because, as mentioned above, the age for Grade III mineralization can be artificially raised by looking at older dogs. Therefore, the end of Grade II mineralization is considered more meaningful to determine the date from which complete mineralization can be expected. Growth times for up to 15 months are described in the literature, and thus, a cut‐off value between 12 and 15 months (13.5 months) was chosen in our study [21].

Additionally, it must be stated that in this article, only the radiographically visible mineralization grade was evaluated without histological confirmation of a specific mineralization grade. Therefore, all assigned grades in this study were radiographically visible mineralization grades. This was considered acceptable, as the study aimed to provide a radiographic classification of the development of rib cartilage mineralization.

## 5 Conclusion

In conclusion, this article is the first detailed description of the age of onset and progression of rib cartilage mineralization in 132 dog breeds. Rib cartilage mineralization times prove to be a reproducible indicator of age in dogs, as age is the primary predictor of mineralization. Although a more comprehensive time range for mineralization is provided, the interpretation of rib cartilage mineralization can aid in determining age in young dogs of unknown origin. Costal cartilage mineralization in our study begins at 71 (Grade II) or 132 days (Grade III), and after 223 days, only complete mineralization was observed. These times could serve as an estimate for when the onset and complete rib cartilage mineralization should be expected. The results in this study are based on extensive and complementary statistical methods and, hence, are therefore to a great extent a reliable approximation of the exact description of the rib cartilage mineralization process. Onset and complete rib cartilage mineralization in our sample occurs earlier in small‐breed dogs and could result mainly from chondrodystrophy rather than the dog's size. Moreover, using logistic regression models in retrospective progress studies of age‐related developments in young animals yields promising results that could be used in practice for age estimation and in further studies.

## Author Contributions

Conception and design: Monika Isabel Hoppe, Carolin Fischer, Mareike‐Kristin Marx, and Sebastian Schaub. Acquisition of data: Monika Isabel Hoppe. Analysis and interpretation of data: Monika Isabel Hoppe, Carolin Fischer, Mareike‐Kristin Marx, and Sebastian Schaub. Drafting the article: Monika Isabel Hoppe. Revising article for intellectual content: Carolin Fischer, Mareike‐Kristin Marx, and Sebastian Schaub. Final approval of the completed article: Monika Isabel Hoppe, Carolin Fischer, Mareike‐Kristin Marx, and Sebastian Schaub. Agreement to be accountable for all aspects of the work in ensuring that questions related to the accuracy and integrity of any part of the work are appropriately investigated and resolved: Monika Isabel Hoppe, Carolin Fischer, Mareike‐Kristin Marx, and Sebastian Schaub.

## Disclosure

The abstract of this article was presented at the ECVDI congress in Athens, Greece in September 2024. No EQUATOR network or other reporting guideline checklist was used.

## Conflicts of Interest

The authors declare no conflicts of interest.

## Supporting information




**Supplementary File 1**: vru70177‐sup‐0001‐Appendix.docx.

## Data Availability

The raw data supporting the conclusions of this article will be made available by the authors, without undue reservation.
