# Supplementary material for: Visual Assessment of Rib Cartilage Mineralization in Thoracic Radiographs as an Indicator of Age in Juvenile Dogs of Various Breeds
Source: Vet Radiol Ultrasound. 2026 Apr 22;67:e70177. doi: 10.1111/vru.70177 (PMC13101407; doi:10.1111/vru.70177)

# Visual assessment of rib cartilage mineralization in thoracic radiographs as an indicator of age in juvenile dogs of various breeds – Appendix

Table of contents

[Rib cartilage mineralization times in juvenile dogs is earlier in small breeds – Appendix 1](#_Toc221815710)

[Table 1: Distribution of breeds and individuals in the study population 2](#_Toc221815711)

[Table 2: Radiographic positions of the individuals 6](#_Toc221815712)

[Table 3: Distribution of gender 6](#_Toc221815713)

[Table 4: Distribution of chondrodystrophy in the five breed groups 7](#_Toc221815714)

[Table 5: Distribution of chondrodystrophy in the three mineralization grades 7](#_Toc221815715)

[Table 6: Grade II mineralization in different breed groups 7](#_Toc221815716)

[Table 6.1: ANOVA 7](#_Toc221815717)

[Table 6.2: ANOVA Effect size 7](#_Toc221815718)

[Table 6.3: ANOVA with Post-Hoc-Test and Bonferroni-Correction – comparison between breed groups 8](#_Toc221815719)

[Table 6.4: Kruskal-Wallis-Test hypothesis for Grade II mineralization 8](#_Toc221815720)

[Table 6.5: Summary of the Kruskal-Wallis test for independent samples 8](#_Toc221815721)

[Table 6.6: Pairwise comparisons with Kruskal-Wallis 9](#_Toc221815722)

[Table 6.7: Effect sizes for Kruskal-Wallis Grade II 9](#_Toc221815723)

[Table 7: Grade III mineralization in different breed groups 9](#_Toc221815724)

[Table 7.1: ANOVA 9](#_Toc221815725)

[Table 7.2: Anova effect size 9](#_Toc221815726)

[Table 7.3: ANOVA with Post-Hoc-Test and Bonferroni-Correction – comparison between breed groups 10](#_Toc221815727)

[7.4: Kruskal-Wallis-Test hypothesis for Grade III mineralization 11](#_Toc221815728)

[7.5: Summary of the Kruskal-Wallis test for independent samples 11](#_Toc221815729)

[7.6: Pairwise comparison of the Kruskal-Wallis 11](#_Toc221815730)

[7.7: Effect sizes for Kruskal-Wallis Grade III 11](#_Toc221815731)

[Table 8: Logistic Regression Grade III – yes/ no 12](#_Toc221815732)

[Table 9: Grade II chondrodystrophic dogs 12](#_Toc221815733)

[Table 9.1: Levene Test for equality of variances and T-Test for equality of means 12](#_Toc221815734)

[Table 9.2: Effect sizes for independent variables 13](#_Toc221815735)

[Table 9.3: Grade II chondrodystrophic dogs Mann-Whitney-U-Test 13](#_Toc221815736)

[Table 9.4: Summary of the Mann-Whitney U-test for independent samples 13](#_Toc221815737)

[Table 10: Grade III chondrodystrophic dogs 13](#_Toc221815738)

[Table 10.1: Levene Test for equality of variances and T-Test for equality of means 14](#_Toc221815739)

[Table 10.2: Effect sizes for independent variables 14](#_Toc221815740)

[Table 10.3: Grade III chondrodystrophic dogs Mann-Whitney-U-Test 14](#_Toc221815741)

[Table 10.4: Summary of the Mann-Whitney U-test for independent samples 15](#_Toc221815742)

[Table 11: Logistic regression analysis of dogs in Grade III 15](#_Toc221815743)

[Table 12: Sex in Grade II mineralization 15](#_Toc221815744)

[Table 12.1: Levene Test for equality of variances and T-Test for equality of means 16](#_Toc221815745)

[Table 12.2: Effect sizes for independent variables 16](#_Toc221815746)

[Table 12.3: Mann-Whitney-U-Test for sex in Grade II 16](#_Toc221815747)

[Table 12.4: Summary of the Mann-Whitney U-test for independent samples 16](#_Toc221815748)

[Table 13: Sex in Grade III mineralization 17](#_Toc221815749)

[Table 13.1: Levene Test for equality of variances and T-Test for equality of means 17](#_Toc221815750)

[Table 13.2: Effect sizes for independent variables 17](#_Toc221815751)

[Table 13.3: Mann-Whitney U Test for sex in Grade III 18](#_Toc221815752)

[Table 13.4: Summary of the Mann-Whitney U-test for independent samples 18](#_Toc221815753)

[Table 14: Kolmogorov-Smirnov-Test for normal distribution of data for Grade II mineralization 18](#_Toc221815754)

[Table 15: Kolmogorov-Smirnov-Test for normal distribution of data for Grade III mineralization 19](#_Toc221815755)

[Table 16: Binary logistic regression analysis with age as the only predictor 20](#_Toc221815756)

[Figure 1: Grade II mineralization in different breed groups – effect size for ANOVA 21](#_Toc221815757)

[Figure 2: Grade III mineralization in different breed groups - effect size for ANOVA 21](#_Toc221815758)

[Figure 3: Grade II chondrodystrophic dogs - effect size for T-Test 21](#_Toc221815759)

[Figure 4: Grade II chondrodystrophic dogs - effect size for effect size for Mann-Whitney U Test 21](#_Toc221815760)

[Figure 5: Grade III chondrodystrophic dogs - effect size for T-Test 21](#_Toc221815761)

[Figure 6: Grade III chondrodystrophic dogs - effect size for Mann-Whitney-U-Test 21](#_Toc221815762)

[Figure 7: Grade II mineralization in different sexes - effect size for T-Test 21](#_Toc221815763)

[Figure 8: Grade II mineralization in different sexes - effect size for Mann-Whitney-U-Test 22](#_Toc221815764)

[Figure 9: QQ Plot of data distribution for Grade II mineralization 22](#_Toc221815765)

[Figure 10: QQ Plot of data distribution for Grade III mineralization 22](#_Toc221815766)

## Table 1: Distribution of breeds and individuals in the study population

| **Dogs** | | | | | |
| --- | --- | --- | --- | --- | --- |
|  | | Quantity (n) | Percentage | Valid percentage | Cumulated percentage |
|  | Mixed Breed | 251 | 19,2 | 19,2 | 19,2 |
|  | German Shepard | 54 | 4,1 | 4,1 | 23,3 |
|  | Bernese Mountain Dog | 17 | 1,3 | 1,3 | 24,6 |
|  | Labrador Retriever | 89 | 6,8 | 6,8 | 31,4 |
|  | Dobermann | 15 | 1,1 | 1,1 | 32,5 |
|  | Dachshund | 35 | 2,7 | 2,7 | 35,2 |
|  | Dalmatian | 6 | ,5 | ,5 | 35,6 |
|  | French Bulldog | 79 | 6,0 | 6,0 | 41,7 |
|  | Golden Retriever | 33 | 2,5 | 2,5 | 44,2 |
|  | Jack Russel Terrier | 19 | 1,5 | 1,5 | 45,6 |
|  | American Staffordshire Terrier | 8 | ,6 | ,6 | 46,3 |
|  | Welsh Springer Spaniel | 1 | ,1 | ,1 | 46,3 |
|  | Parson Russel Terrier | 4 | ,3 | ,3 | 46,6 |
|  | Bull Terrier | 6 | ,5 | ,5 | 47,1 |
|  | German Pinscher | 10 | ,8 | ,8 | 47,9 |
|  | Boston Terrier | 5 | ,4 | ,4 | 48,2 |
|  | Mastino Neapolitano | 1 | ,1 | ,1 | 48,3 |
|  | Flat Coated Retriever | 6 | ,5 | ,5 | 48,8 |
|  | Boxer | 28 | 2,1 | 2,1 | 50,9 |
|  | English Bulldog | 24 | 1,8 | 1,8 | 52,7 |
|  | Dutch Shepard Dog | 2 | ,2 | ,2 | 52,9 |
|  | Chihuahua | 62 | 4,7 | 4,7 | 57,6 |
|  | German Hunting Terrier | 2 | ,2 | ,2 | 57,8 |
|  | Bearded Collie | 3 | ,2 | ,2 | 58,0 |
|  | Hovawart | 7 | ,5 | ,5 | 58,5 |
|  | Miniature Pinscher | 5 | ,4 | ,4 | 58,9 |
|  | Airedale Terrier | 3 | ,2 | ,2 | 59,2 |
|  | German Wirehaired Pointer | 6 | ,5 | ,5 | 59,6 |
|  | Malinois | 8 | ,6 | ,6 | 60,2 |
|  | Weimaraner | 6 | ,5 | ,5 | 60,7 |
|  | Pug | 19 | 1,5 | 1,5 | 62,1 |
|  | Border Terrier | 7 | ,5 | ,5 | 62,7 |
|  | Husky | 8 | ,6 | ,6 | 63,3 |
|  | Cane Corso | 3 | ,2 | ,2 | 63,5 |
|  | Swiss Mountain Dog | 8 | ,6 | ,6 | 64,1 |
|  | Samoyed Dog | 1 | ,1 | ,1 | 64,2 |
|  | Yorkshire Terrier | 19 | 1,5 | 1,5 | 65,6 |
|  | Pitbull | 4 | ,3 | ,3 | 66,0 |
|  | Bolonka Zwetna | 11 | ,8 | ,8 | 66,8 |
|  | Basset Hound | 1 | ,1 | ,1 | 66,9 |
|  | Tibet Terrier | 1 | ,1 | ,1 | 66,9 |
|  | Alaskan Malamute | 2 | ,2 | ,2 | 67,1 |
|  | Entlebuch Mountain Dog | 8 | ,6 | ,6 | 67,7 |
|  | Saarloos Wolfdog | 3 | ,2 | ,2 | 67,9 |
|  | Border Collie | 23 | 1,8 | 1,8 | 69,7 |
|  | Bullmastiff | 3 | ,2 | ,2 | 69,9 |
|  | German Spaniel | 9 | ,7 | ,7 | 70,6 |
|  | Rhodesian Ridgeback | 13 | 1,0 | 1,0 | 71,6 |
|  | Kleiner Münsterländer | 4 | ,3 | ,3 | 71,9 |
|  | Maltese Dog | 20 | 1,5 | 1,5 | 73,4 |
|  | Irish Setter | 13 | 1,0 | 1,0 | 74,4 |
|  | Collie | 7 | ,5 | ,5 | 74,9 |
|  | Great Dane | 10 | ,8 | ,8 | 75,7 |
|  | Bouvier de Flandres | 1 | ,1 | ,1 | 75,8 |
|  | Australian Shepard | 36 | 2,7 | 2,7 | 78,5 |
|  | Cocker Spaniel | 9 | ,7 | ,7 | 79,2 |
|  | Fox Terrier | 4 | ,3 | ,3 | 79,5 |
|  | Spanish Water Dog | 1 | ,1 | ,1 | 79,6 |
|  | Magyar Vizsla | 10 | ,8 | ,8 | 80,4 |
|  | Landseer | 4 | ,3 | ,3 | 80,7 |
|  | Rottweiler | 8 | ,6 | ,6 | 81,3 |
|  | Tervuren Dog | 1 | ,1 | ,1 | 81,4 |
|  | Beagle | 12 | ,9 | ,9 | 82,3 |
|  | German Shorthaired Pointer | 3 | ,2 | ,2 | 82,5 |
|  | Leonberger | 3 | ,2 | ,2 | 82,7 |
|  | American Bulldog | 2 | ,2 | ,2 | 82,9 |
|  | Berger Blanc Swiss | 11 | ,8 | ,8 | 83,7 |
|  | Dogo Canario | 1 | ,1 | ,1 | 83,8 |
|  | Miniature Bullterrier | 2 | ,2 | ,2 | 84,0 |
|  | Havanese | 14 | 1,1 | 1,1 | 85,0 |
|  | Chinese Crested | 1 | ,1 | ,1 | 85,1 |
|  | Australian Terrier | 1 | ,1 | ,1 | 85,2 |
|  | Australian Cattle Dog | 1 | ,1 | ,1 | 85,3 |
|  | Gos d´Atura Catalá | 1 | ,1 | ,1 | 85,3 |
|  | Eurasian | 5 | ,4 | ,4 | 85,7 |
|  | Rusky Toy | 4 | ,3 | ,3 | 86,0 |
|  | Appenezeller Mountain Dog | 5 | ,4 | ,4 | 86,4 |
|  | Shitzu | 11 | ,8 | ,8 | 87,3 |
|  | Anatolian Shepard Dog | 1 | ,1 | ,1 | 87,3 |
|  | Bichon Frisé | 5 | ,4 | ,4 | 87,7 |
|  | Russian Terrier | 1 | ,1 | ,1 | 87,8 |
|  | West Highland White Terrier | 18 | 1,4 | 1,4 | 89,2 |
|  | Basset Fauvre de Bretange | 1 | ,1 | ,1 | 89,2 |
|  | English Springer Spaniel | 1 | ,1 | ,1 | 89,3 |
|  | Czechoslovakian Wolfdog | 3 | ,2 | ,2 | 89,5 |
|  | Gordon Setter | 2 | ,2 | ,2 | 89,7 |
|  | St. Bernard | 3 | ,2 | ,2 | 89,9 |
|  | Austrian Black and Tan Hound | 3 | ,2 | ,2 | 90,2 |
|  | Shiba Inu | 5 | ,4 | ,4 | 90,5 |
|  | Polski Owczarek Nizinny | 9 | ,7 | ,7 | 91,2 |
|  | Bavarian Mountain Dog | 5 | ,4 | ,4 | 91,6 |
|  | Styrian Wirehaired Hound | 1 | ,1 | ,1 | 91,7 |
|  | Groenendale | 2 | ,2 | ,2 | 91,8 |
|  | Sheltie | 16 | 1,2 | 1,2 | 93,1 |
|  | Bobtail | 4 | ,3 | ,3 | 93,4 |
|  | Welsh Corgi Pembroke | 9 | ,7 | ,7 | 94,0 |
|  | Pointer | 2 | ,2 | ,2 | 94,2 |
|  | Cavalier King Charles Spaniel | 16 | 1,2 | 1,2 | 95,4 |
|  | Irish Wolfhound | 2 | ,2 | ,2 | 95,6 |
|  | Great Münsterländer | 1 | ,1 | ,1 | 95,6 |
|  | Hanoverian bloodhound | 1 | ,1 | ,1 | 95,7 |
|  | Scottish Terrier | 1 | ,1 | ,1 | 95,8 |
|  | Italian Greyhound | 5 | ,4 | ,4 | 96,2 |
|  | Akita Inu | 2 | ,2 | ,2 | 96,3 |
|  | Whippet | 2 | ,2 | ,2 | 96,5 |
|  | Barsoi | 2 | ,2 | ,2 | 96,6 |
|  | Norwich Terrier | 1 | ,1 | ,1 | 96,7 |
|  | Kangal | 1 | ,1 | ,1 | 96,8 |
|  | Lagotto Romagnolo | 1 | ,1 | ,1 | 96,9 |
|  | Newfoundland dog | 6 | ,5 | ,5 | 97,3 |
|  | Papillion | 3 | ,2 | ,2 | 97,6 |
|  | Portuguese Waterdog | 1 | ,1 | ,1 | 97,6 |
|  | Bordeaux Mastiff | 2 | ,2 | ,2 | 97,8 |
|  | Chow Chow | 1 | ,1 | ,1 | 97,9 |
|  | Coton de Tulear | 1 | ,1 | ,1 | 97,9 |
|  | Schapendoes | 2 | ,2 | ,2 | 98,1 |
|  | Berge de Pyrenees | 2 | ,2 | ,2 | 98,2 |
|  | Japanese Chin | 1 | ,1 | ,1 | 98,3 |
|  | Crain Terrier | 3 | ,2 | ,2 | 98,5 |
|  | Welsh Terrier | 1 | ,1 | ,1 | 98,6 |
|  | Tibetan Mastiff | 1 | ,1 | ,1 | 98,7 |
|  | Kooikerhond | 1 | ,1 | ,1 | 98,8 |
|  | German Long Haired Pointer | 2 | ,2 | ,2 | 98,9 |
|  | Bolognese dog | 2 | ,2 | ,2 | 99,1 |
|  | Shar Pai | 1 | ,1 | ,1 | 99,2 |
|  | Harz fox | 1 | ,1 | ,1 | 99,2 |
|  | Griffon | 1 | ,1 | ,1 | 99,3 |
|  | Austrian Pinscher | 2 | ,2 | ,2 | 99,5 |
|  | Löwchen | 1 | ,1 | ,1 | 99,5 |
|  | Podenco Iberico | 2 | ,2 | ,2 | 99,7 |
|  | Galgo Español | 1 | ,1 | ,1 | 99,8 |
|  | German Hound | 3 | ,2 | ,2 | 100,0 |
|  | Total | 1310 | 100,0 | 100,0 |  |

## Table 2: Radiographic positions of the individuals

1.right lateral, 2.left lateral, 3.ventrodorsal, 4.dorsoventral, 5.survey right laterolateral, 6.survey ventrodorsal, 7. survey left laterolateral

| Radiographic position | Quantity (n) | Percentage | Valid Percentage | Cumulative Percentage |
| --- | --- | --- | --- | --- |
| 1 | 160 | 12,24 | 12,24 | 12,24 |
| 1,2 | 2 | 0,20 | 0,20 | 12,44 |
| 1,2,3,4 | 1 | 0,10 | 0,10 | 12,54 |
| 1,2,4 | 2 | 0,20 | 0,20 | 12,74 |
| 1,3 | 298 | 22,69 | 22,69 | 35,42 |
| 1,4 | 815 | 61,99 | 61,99 | 97,41 |
| 1,5 | 2 | 0,20 | 0,20 | 97,61 |
| 5,4 | 3 | 0,30 | 0,30 | 97,91 |
| 5 | 1 | 0,10 | 0,10 | 98,01 |
| 5,3 | 12 | 0,90 | 0,90 | 98,91 |
| 5,6 | 1 | 0,10 | 0,10 | 99,00 |
| 7,4 | 13 | 1,00 | 1,00 | 100,00 |
|  | 1310 | 100 | 100 |  |

## Table 3: Distribution of gender

|  | | Male | Female |  |
| --- | --- | --- | --- | --- |
| Breed Group | Small | 97 | 88 | 185 |
|  | Toy | 56 | 77 | 133 |
|  | Medium | 166 | 140 | 306 |
|  | Large | 174 | 193 | 367 |
|  | Giant | 33 | 35 | 68 |
|  | Mixed | 122 | 129 | 251 |
| total | | 648 | 662 | 1310 |

## Table 4: Distribution of chondrodystrophy in the five breed groups

|  | | Non-Chondrodystrophic. | Chondrodystrophic | Sum |
| --- | --- | --- | --- | --- |
| Breed Group | Small | 47 | 138 | 185 |
|  | Toy | 83 | 50 | 133 |
|  | Medium | 166 | 140 | 306 |
|  | Large | 367 | 0 | 367 |
|  | Giant | 68 | 0 | 68 |
| total | | 731 | 328 | 1059 |

## Table 5: Distribution of chondrodystrophy in the three mineralization grades

|  | | Non-Condrodystrophic | Chondrodystrophic | Sum |
| --- | --- | --- | --- | --- |
| Grade | 1,00 | 303 | 102 | 405 |
|  | 2,00 | 274 | 138 | 412 |
|  | 3,00 | 154 | 88 | 242 |
| total | | 731 | 328 | 1059 |

| Table 6: Grade II mineralization in different breed groupsTable 6.1: ANOVA | | | | | |
| --- | --- | --- | --- | --- | --- |
|  | | | | | |
|  | Sum of Squares | df | Mean square | F | Sig. |
| Between groups | 36581,399 | 4 | 9145,350 | 9,843 | ,000 |
| Within one group | 378139,676 | 407 | 929,090 |  |  |
| General | 414721,075 | 411 |  |  |  |

### Table 6.2: ANOVA Effect size

|  | | | | |
| --- | --- | --- | --- | --- |
|  | | Point estimate | 95% confidence interval | |
|  |  |  | Lower bounds | Upper bounds |
| Age for grade II mineralization | Eta-Square | ,088 | ,037 | ,136 |
|  | Epsilon-Square | ,079 | ,027 | ,128 |
|  | Omega-Square, fixed effects | ,079 | ,027 | ,128 |
|  | Omega-Quadrat, random effects | ,021 | ,007 | ,035 |
| a. Eta-squared and epsilon-squared are estimated based on the fixed effects model. | | | | |

### Table 6.3: ANOVA with Post-Hoc-Test and Bonferroni-Correction – comparison between breed groups

| (I) Dog breed group | (J) breed groups for comparison | Mean difference (I-J) | Standard error | Significance | 95% confidence interval | |
| --- | --- | --- | --- | --- | --- | --- |
|  |  |  |  |  | Lower bounds | Upper Bounds |
| small | toy | -5,02372 | 5,24807 | 1,000 | -19,8360 | 9,7885 |
|  | medium | -14,80157^*^ | 4,52225 | ,012 | -27,5653 | -2,0379 |
|  | big | -23,08330^*^ | 4,61222 | ,000 | -36,1009 | -10,0657 |
|  | giant | -31,71826^*^ | 7,11538 | ,000 | -51,8009 | -11,6356 |
| toy | small | 5,02372 | 5,24807 | 1,000 | -9,7885 | 19,8360 |
|  | medium | -9,77785 | 4,58942 | ,337 | -22,7311 | 3,1754 |
|  | big | -18,05959^*^ | 4,67810 | ,001 | -31,2632 | -4,8560 |
|  | giant | -26,69455^*^ | 7,15826 | ,002 | -46,8982 | -6,4909 |
| medium | small | 14,80157^*^ | 4,52225 | ,012 | 2,0379 | 27,5653 |
|  | toy | 9,77785 | 4,58942 | ,337 | -3,1754 | 22,7311 |
|  | big | -8,28173 | 3,84618 | ,319 | -19,1373 | 2,5738 |
|  | giant | -16,91669 | 6,64449 | ,113 | -35,6702 | 1,8369 |
| big | small | 23,08330^*^ | 4,61222 | ,000 | 10,0657 | 36,1009 |
|  | toy | 18,05959^*^ | 4,67810 | ,001 | 4,8560 | 31,2632 |
|  | medium | 8,28173 | 3,84618 | ,319 | -2,5738 | 19,1373 |
|  | giant | -8,63496 | 6,70605 | 1,000 | -27,5623 | 10,2923 |
| giant | small | 31,71826^*^ | 7,11538 | ,000 | 11,6356 | 51,8009 |
|  | toy | 26,69455^*^ | 7,15826 | ,002 | 6,4909 | 46,8982 |
|  | medium | 16,91669 | 6,64449 | ,113 | -1,8369 | 35,6702 |
|  | big | 8,63496 | 6,70605 | 1,000 | -10,2923 | 27,5623 |
| *. The mean difference is significant at level 0.05. | | | | | | |

| Table 6.4: Kruskal-Wallis-Test hypothesis for Grade II mineralization | | | | |
| --- | --- | --- | --- | --- |
|  | Null hypothesis | Test | Sig.^a,b^ | Decision |
| 1 | The onset of mineralization is identical across all breed categories | Kruskal-Wallis-Test for independent samples | ,000 | Reject null hypothesis |

a. The significance level is 0.50

b. Asymptotic significance is indicated

| Table 6.5: Summary of the Kruskal-Wallis test for independent samples | |
| --- | --- |
| Total number | 412 |
| Test statistics | 36,369^a^ |
| Degree of freedom | 4 |
| Asymptotic Sig. (two-sided test) | ,000 |
| a. The test statistics are adjusted for bindings | |

| Table 6.6: Pairwise comparisons with Kruskal-Wallis | | | | | |
| --- | --- | --- | --- | --- | --- |
| Sample 1-Sample 2 | Test statistics | Standard error | Standard test statistics | Significance | Adjusted significance |
| small-toy | -13,337 | 20,501 | -,651 | ,515 | 1,000 |
| small-medium | -54,939 | 17,665 | -3,110 | ,002 | ,019 |
| small-large | -85,063 | 18,017 | -4,721 | ,000 | ,000 |
| small-giant | -115,020 | 27,795 | -4,138 | ,000 | ,000 |
| toy-medium | -41,602 | 17,928 | -2,321 | ,020 | ,203 |
| toy-large | -71,726 | 18,274 | -3,925 | ,000 | ,001 |
| toy-giant | -101,683 | 27,963 | -3,636 | ,000 | ,003 |
| medium-large | -30,124 | 15,025 | -2,005 | ,045 | ,450 |
| medium-giant | -60,081 | 25,956 | -2,315 | ,021 | ,206 |
| big-giant | -29,957 | 26,196 | -1,144 | ,253 | 1,000 |
| Each row tests the null hypothesis (same distributions in sample 1 and sample 2). | | | | | |
| Asymptotic significances (two-sided tests) are displayed. The significance level is 0.50. | | | | | |

### Table 6.7: Effect sizes for Kruskal-Wallis Grade II

| Small vs. | medium | large | giant |
| --- | --- | --- | --- |
|  | $r=\left\vert\frac{-3,110}{\sqrt{69+66}}\right\vert=0,268$ | $r=\left\vert\frac{-4,721}{\sqrt{69+119}}\right\vert=0,344$ | $r=\left\vert\frac{-4,138}{\sqrt{69+25}}\right\vert=0,427$ |
| Toy vs. | $r=\left\vert\frac{-2,321}{\sqrt{66+133}}\right\vert=0,165$ | $r=\left\vert\frac{-3,925}{\sqrt{66+119}}\right\vert=0,289$ | $r=\left\vert\frac{-3,636}{\sqrt{66+25}}\right\vert=0,381$ |

## Table 7: Grade III mineralization in different breed groups

### Table 7.1: ANOVA

|  | Sum of squares | df | Mean square | F | Sig. |
| --- | --- | --- | --- | --- | --- |
| Between Groups | 72447,839 | 4 | 18111,960 | 4,528 | ,002 |
| Within one group | 948101,929 | 237 | 4000,430 |  |  |
| Total | 1020549,769 | 241 |  |  |  |

### Table 7.2: Anova effect size

|  | | | | |
| --- | --- | --- | --- | --- |
|  | | Point estimate | 95% confidence interval | |
|  |  |  | Lower Bonds | Upper Bonds |
| Age for grade II mineralization | Eta-Square | ,071 | ,012 | ,128 |
|  | Epsilon-Square | ,055 | -,005 | ,113 |
|  | Omega-Square, fixed effect | ,055 | -,005 | ,113 |
|  | Omega-Square, random effect | ,014 | -,001 | ,031 |

a. Eta-squared and epsilon-squared are estimated based on the fixed effects model.

b. Negative but less biased estimates are retained, not rounded up to zero.

### Table 7.3: ANOVA with Post-Hoc-Test and Bonferroni-Correction – comparison between breed groups

| (I) Dog breed group | (J) Breed group for comparision | Mean difference (I-J) | Standard error | Significance | 95% confidence interval | |
| --- | --- | --- | --- | --- | --- | --- |
|  |  |  |  |  | Lower bounds | Upper Bounds |
| small | toy | -28,04670 | 14,82578 | ,597 | -70,0564 | 13,9630 |
|  | medium | -40,18242^*^ | 11,57931 | ,006 | -72,9930 | -7,3718 |
|  | big | -35,41359^*^ | 11,35275 | ,020 | -67,5822 | -3,2449 |
|  | giant | -62,68718^*^ | 18,53717 | ,008 | -115,2133 | -10,1611 |
| toy | small | 28,04670 | 14,82578 | ,597 | -13,9630 | 70,0564 |
|  | medium | -12,13571 | 14,14290 | 1,000 | -52,2104 | 27,9390 |
|  | big | -7,36688 | 13,95801 | 1,000 | -46,9177 | 32,1839 |
|  | giant | -34,64048 | 20,23778 | ,883 | -91,9854 | 22,7044 |
| medium | small | 40,18242^*^ | 11,57931 | ,006 | 7,3718 | 72,9930 |
|  | toy | 12,13571 | 14,14290 | 1,000 | -27,9390 | 52,2104 |
|  | big | 4,76883 | 10,44522 | 1,000 | -24,8283 | 34,3660 |
|  | giant | -22,50476 | 17,99568 | 1,000 | -73,4965 | 28,4870 |
| big | small | 35,41359^*^ | 11,35275 | ,020 | 3,2449 | 67,5822 |
|  | toy | 7,36688 | 13,95801 | 1,000 | -32,1839 | 46,9177 |
|  | medium | -4,76883 | 10,44522 | 1,000 | -34,3660 | 24,8283 |
|  | giant | -27,27359 | 17,85074 | 1,000 | -77,8547 | 23,3075 |
| giant | small | 62,68718^*^ | 18,53717 | ,008 | 10,1611 | 115,2133 |
|  | toy | 34,64048 | 20,23778 | ,883 | -22,7044 | 91,9854 |
|  | medium | 22,50476 | 17,99568 | 1,000 | -28,4870 | 73,4965 |
|  | big | 27,27359 | 17,85074 | 1,000 | -23,3075 | 77,8547 |
| *. The mean difference is significant at level 0.05. | | | | | | |

| 7.4: Kruskal-Wallis-Test hypothesis for Grade III mineralization | | | | |
| --- | --- | --- | --- | --- |
|  | Null hypothesis | Test | Sig.^a,b^ | Decision |
| 1 | The time for full mineralization is identical across all breed categories | Kruskal-Wallis-Test for independent samples | ,000 | Reject null hypothesis |

a. The significance level is 0.50

b. Asymptotic significance is indicated

| 7.5: Summary of the Kruskal-Wallis test for independent samples | |
| --- | --- |
| Total number | 242 |
| Test statistics | 25,179^a^ |
| Degree of freedom | 4 |
| Asymptotic Sig. (two-sided test) | ,000 |
| a. The test statistics are adjusted for bindings | |

| 7.6: Pairwise comparison of the Kruskal-Wallis | | | | | |
| --- | --- | --- | --- | --- | --- |
| Sample 1-Sample 2 | Test statistics | Standard error | Standard test statistics | Significane | Adjustated significance |
| small-toy | -32,523 | 16,408 | -1,982 | ,047 | ,475 |
| small-large | -43,903 | 12,564 | -3,494 | ,000 | ,005 |
| small-medium | -53,402 | 12,815 | -4,167 | ,000 | ,000 |
| small-giant | -81,035 | 20,515 | -3,950 | ,000 | ,001 |
| toy-large | -11,380 | 15,447 | -,737 | ,461 | 1,000 |
| toy-medium | -20,879 | 15,652 | -1,334 | ,182 | 1,000 |
| toy-giant | -48,512 | 22,397 | -2,166 | ,030 | ,303 |
| big-medium | 9,499 | 11,560 | ,822 | ,411 | 1,000 |
| big-giant | -37,132 | 19,755 | -1,880 | ,060 | ,602 |
| medium-giant | -27,633 | 19,916 | -1,388 | ,165 | 1,000 |
| Each row tests the null hypothesis (same distributions in sample 1 and sample 2). | | | | | |
| Asymptotic significances (two-sided tests) are displayed. The significance level is 0.50. | | | | | |

### 7.7: Effect sizes for Kruskal-Wallis Grade III

| Group 1 versus Group 2 | Standardized test statistics z | Significance | N1 /N2 | Effect size r |
| --- | --- | --- | --- | --- |
| Small/ Medium | -4,167 | 0,005 | 52/70 | 0,376 |
| Small / Large | -3,494 | < 0,001 | 52/77 | 0,315 |
| Small/ Giant | -3,950 | 0,001 | 52/15 | 0,481 |

## Table 8: Logistic Regression Grade III – yes/ no

Various predictor combinations age, weight, gender, chondrodystrophy

| Predictors | χ^2^ (df)/  Wald | p | n | Exp(B)  Odds | % right | B_i_ | -const. |
| --- | --- | --- | --- | --- | --- | --- | --- |
| All breeds | 704,7(2) | <0.001 | 1059 |  | 89,0 |  | 12,24 |
| Age | 155,6 | <0.001 |  | 1,065 |  | 0,063 |  |
| Sex | 2,0 | 0.155 |  | 1,188 |  | 0,173 |  |
| All breeds | 665,9(2) | <0.001 | 996 |  | 89,0 |  | 12,24 |
| Age | 155,6 | <0.001 |  | 1,070 |  | 0,067 | 12,09 |
| Weight | 2,0 | 0.001 |  | 0,951 |  | -0,050 |  |
| All breeds | 718,9(2) | <0.001 | 1059 |  | 89,8 |  | 12,24 |
| Age | 154,6 | <0.001 |  | 1,067 |  | 0,065 | 12,70 |
| Chondro. | 15,6 | <0.001 |  | 2,837 |  | 1,043 |  |
| All breeds | 721,4(3) | <0.001 | 1059 |  | 89,5 |  | 13,14 |
| Age | 154,6 | <0.001 |  | 1,068 |  | 0,065 |  |
| Sex | 2,5 | 0,115 |  | 1,216 |  | 0,196 |  |
| Chondro. | 16,1 | <0.001 |  | 2,891 |  | 1,062 |  |
| All breeds | 671,1(3) | <0.001 | 996 |  | 89,6 |  | 12,53 |
| Age | 145,8 | <0.001 |  | 1,070 |  | 0,067 |  |
| Weight | 4,2 | 0,041 |  | 0,968 |  | -0,033 |  |
| Chondro. | 5,2 | 0.022 |  | 1,983 |  | 0,690 |  |

## Table 9: Grade II chondrodystrophic dogs

| Group statistic | | | | | |
| --- | --- | --- | --- | --- | --- |
|  | Chondrodystrophic (1)  Non – Chondrodystrophic (0) | n | mean | Standard error | Standard error of the mean |
| Age of Grade II mineralization | ,00 | 274 | 152,4307 | 31,20141 | 1,88495 |
|  | 1,00 | 138 | 138,2971 | 30,87706 | 2,62843 |

| Table 9.1: Levene Test for equality of variances and T-Test for equality of means | | | | | | | | | | |
| --- | --- | --- | --- | --- | --- | --- | --- | --- | --- | --- |
|  | | Levene | | T-Test | | | | | | |
|  |  | F | Sig. | T | df | Sig. (2-sided) | Mean difference | Standard error difference | 95% confidence interval of the difference | |
|  |  |  |  |  |  |  |  |  | Lower Value | Upper Value |
| Age of Grade II mineralization | Variances are equal | **,282** | **,596** | **4,355** | **410** | **,000** | **14,13356** | **3,24565** | **7,75336** | **20,51375** |
|  | Variances are not equal |  |  | 4,370 | 277,339 | ,000 | 14,13356 | 3,23445 | 7,76636 | 20,50075 |

| Table 9.2: Effect sizes for independent variables | | | | | |
| --- | --- | --- | --- | --- | --- |
|  | | **Standardizer^a^** | Point estimate | 95% confidence interval | |
|  |  |  |  | Lower bounds | Upper Bounds |
| Age of grade II mineraliziation | Cohen's d | 31,09341 | ,455 | ,247 | ,661 |
|  | Hedges' correction | 31,15043 | ,454 | ,247 | ,660 |
|  | Glass' Delta | 30,87706 | ,458 | ,245 | ,669 |
| a. The denominator used in the estimation of the effect sizes.  Cohen's d uses the summarized standard deviation.  Hedges' correction uses the summarized standard deviation and a correction factor.  Glass' delta uses the standard deviation of a sample from the control group | | | | | |

### Table 9.3: Grade II chondrodystrophic dogs Mann-Whitney-U-Test

| Hypothesis test overview | | | | | | |
| --- | --- | --- | --- | --- | --- | --- |
|  | Null hypothesis | | Test | | Sig.^a,b^ | Decicion |
| 1 | The distribution of onset of mineralization (Grade II) is identical across the categories of Chondrodystrophic and non-chondrodystrophic dogs | | Mann-Whitney-U-Test for independent variables | | ,000 | Decline null hypothesis |
| a. The significance level is 0.50. | | | | | | |
| b. Asymptotic significance is indicated. | | | | | | |
| Table 9.4: Summary of the Mann-Whitney U-test for independent samples | | | | | | |
| Total | | 412 | |  |  |  |
| Mann-Whitney-U-Test | | 14286,000 | |  |  |  |
| Wilcoxon-W | | 23877,000 | |  |  |  |
| Test statistics | | 14286,000 | |  |  |  |
| Standard error | | 1140,684 | |  |  |  |
| Standardised test statistics | | -4,050 | |  |  |  |
| Asymptotic Sig. (two-sided test) | | ,000 | |  |  |  |

## Table 10: Grade III chondrodystrophic dogs

| Group statistic | | | | | |
| --- | --- | --- | --- | --- | --- |
|  | Chondrodystrophic (1)  Non – Chondrodystrophic (0) | n | mean | Standard error | Standard error of the mean |
| Age of Grade III mineralization | ,00 | 154 | 242,1169 | 63,18558 | 5,09164 |
|  | 1,00 | 88 | 227,6818 | 67,64011 | 7,21046 |

### Table 10.1: Levene Test for equality of variances and T-Test for equality of means

|  | | | | | | | | | | |
| --- | --- | --- | --- | --- | --- | --- | --- | --- | --- | --- |
|  | | Levene | | T-Test | | | | | | |
|  |  | F | Sig. | T | df | Sig. (2-sided) | Mean difference | Standard error difference | 95% confidence interval of the difference | |
|  |  |  |  |  |  |  |  |  | Lower Value | Upper Value |
| Age of Grade III mineralization | Variances are equal | **,253** | **,615** | **1,666** | **240** | **,097** | **14,43506** | **8,66404** | **-2,63220** | **31,50233** |
|  | Variances are not equal |  |  | 1,635 | 171,192 | ,104 | 14,43506 | 8,82698 | -2,98867 | 31,85880 |

| Table 10.2: Effect sizes for independent variables | | | | | |
| --- | --- | --- | --- | --- | --- |
|  | | **Standardizer^a^** | Point estimate | 95% confidence interval | |
|  |  |  |  | Lower bounds | Upper Bounds |
| Age of Grade III mineralization | Cohen's d | 64,83572 | ,223 | -,040 | ,485 |
|  | Hedges' correction | 65,03922 | ,222 | -,040 | ,484 |
|  | Glass' Delta | 67,64011 | ,213 | -,051 | ,477 |
| a. The denominator used in the estimation of the effect sizes.  Cohen's d uses the summarized standard deviation.  Hedges' correction uses the summarized standard deviation and a correction factor.  Glass' delta uses the standard deviation of a sample from the control group | | | | | |

### Table 10.3: Grade III chondrodystrophic dogs Mann-Whitney-U-Test

| Hypothesis test overview | | | | |
| --- | --- | --- | --- | --- |
|  | Null hypothesis | Test | Significance | Decision |
| 1 | The distribution of the age of full mineralization is identical across the categories of chondrodystrophic and non-chondrodystrophic dogs | Mann-Whitney U-test for independent samples | ,023 | Nullhypothese ablehnen |
| a. The significance level is .050. | | | | |
| b. Asymptotic significance is indicated. | | | | |

| Table 10.4: Summary of the Mann-Whitney U-test for independent samples | |
| --- | --- |
| Total | 242 |
| Mann-Whitney-U-Test | 5584,000 |
| Wilcoxon-W | 9500,000 |
| Test statistics | 5584,000 |
| Standard error | 523,811 |
| Standardized test statistic | -2,276 |
| Asymptotic Sig. (two-sided test) | ,023 |

## Table 11: Logistic regression analysis of dogs in Grade III

| Breed | χ^2^ | p | n | Exp(B) | % | t0.5 | t0.9 | B | -const. |
| --- | --- | --- | --- | --- | --- | --- | --- | --- | --- |
| All breeds | 702,7 | <0.001 | 1059 | 1,064 | 89,1 | 191 | 226 | 0,062 | 11,86 |
| Toy | 80,4 | <0.00 1 | 133 | 1,058 | 89,5 | 191 | 230 | 0,056 | 10,69 |
| Small | 129,5 | <0.001 | 185 | 1,065 | 90,3 | 171 | 205 | 0,063 | 10,76 |
| Medium | 231,0 | <0.001 | 306 | 1,058 | 91,2 | 192 | 217 | 0,085 | 16,30 |
| Large | 237,4 | <0.001 | 367 | 1,065 | 89,1 | 194 | 229 | 0,063 | 12,22 |
| Giant | 53,7 | <0.001 | 68 | 1,101 | 92,6 | 207 | 230 | 0,096 | 19,91 |
| Chondrodyst. | 241,7 | <0.001 | 328 | 1,076 | 90,5 | 180 | 209 | 0,073 | 13,11 |
| Not Chondrodyst. | 474,2 | <0.001 | 731 | 1,063 | 89,5 | 196 | 232 | 0,061 | 11,97 |
| Small Chondrodyst. | 100,9 | <0.001 | 138 | 1,067 | 90,6 | 166 | 199 | 0,065 | 10,76 |
| Small Non-Chondro. | 31,7 | <0.001 | 57 | 1,070 | 89,4 | 183 | 215 | 0,068 | 12,44 |
| Medium Chondrodyst. | 111,6 | <0.001 | 140 | 1,110 | 90,7 | 188 | 209 | 0,104 | 19,53 |
| Medium Non-Chondro. | 120,7 | <0.001 | 160 | 1,079 | 80,0 | 196 | 225 | 0,076 | 14,92 |
| Toy Chondrodyst. | 48,4 | <0.001* | 50 | 1,187 | 98,0 | 180 | 192 | 0,171 | 30,68 |
| Toy Non-Chondro. | 39,83 | <0.001 | 83 | 1,042 | 84,3 | 205 | 259 | 0,041 | 8,42 |

*)Wald Test non significant for B (p=0,056) and Const. (p=0,062)

## Table 12: Sex in Grade II mineralization

| Group statistics | | | | | |
| --- | --- | --- | --- | --- | --- |
|  | Male (1) / Female (3) | n | mean | Standard deviation | Standard error of the mean value |
| Age of Grade II mineralization | 1 | 192 | 153,3542 | 30,47460 | 2,19931 |
|  | 3 | 220 | 142,7591 | 32,11212 | 2,16500 |

| Table 12.1: Levene Test for equality of variances and T-Test for equality of means  \|  \| \| \| \| \| \| \| \| \| \| \| --- \| --- \| --- \| --- \| --- \| --- \| --- \| --- \| --- \| --- \| \|  \| Levene \| \| T-Test \| \| \| \| \| \| \| \| F \| Sig. \| T \| df \| Sig. (2-sided) \| Mean difference \| Standard error difference \| 95% confidence interval of the difference \| \| \| Lower Value \| Upper Value \| | | | | | | | | | | |
| --- | --- | --- | --- | --- | --- | --- | --- | --- | --- | --- | --- | --- | --- | --- | --- | --- | --- | --- | --- | --- | --- | --- | --- | --- | --- | --- | --- | --- | --- | --- | --- | --- | --- | --- | --- | --- | --- | --- | --- | --- | --- |
| Age of Grade II mineralization | Variances are equal | ,639 | ,424 | 3,421 | 410 | ,001 | 10,59508 | 3,09714 | 4,50681 | 16,68334 |
|  | Variances are not equal |  |  | 3,433 | 407,113 | ,001 | 10,59508 | 3,08613 | 4,52833 | 16,66182 |

| Table 12.2: Effect sizes for independent variables | | | | | |
| --- | --- | --- | --- | --- | --- |
|  | | **Standardisierer^a^** | Point estimate | 95% confidence interval | |
|  |  |  |  | Lower bounds | Upper Bounds |
| Age of Grade II mineralization | Cohen's d | 31,35991 | ,338 | ,143 | ,533 |
|  | Hedges' correction | 31,41743 | ,337 | ,142 | ,532 |
|  | Glass' Delta | 32,11212 | ,330 | ,134 | ,526 |
| a. The denominator used in the estimation of the effect sizes.  Cohen's d uses the summarized standard deviation.  Hedges' correction uses the summarized standard deviation and a correction factor.  Glass' delta uses the standard deviation of a sample from the control group. | | | | | |

### Table 12.3: Mann-Whitney-U-Test for sex in Grade II

| Hypothesis test overview | | | | |
| --- | --- | --- | --- | --- |
|  | Null hypothesis | Test | Sig.^a,b^ | Decision |
| 1 | The distribution of Grade II mineralization is identical for both sexes. | Mann-Whitney U-test for independent samples | ,000 | Decline null hypothesis |
| a. The significance level is .050. | | | | |
| b. Asymptotic significance is indicated. | | | | |

| Table 12.4: Summary of the Mann-Whitney U-test for independent samples | |
| --- | --- |
| Total | 412 |
| Mann-Whitney-U-Test | 16751,500 |
| Wilcoxon-W | 41061,500 |
| Test statistics | 16751,500 |
| Standard error | 1205,626 |
| Standardised test statistics | -3,623 |
| Asymptotic Sig. (two-sided test) | ,000 |

## Table 13: Sex in Grade III mineralization

| Group statistics | | | | | |
| --- | --- | --- | --- | --- | --- |
|  | Male (1) / Female (3) | n | mean | Standard deviation | Standard error of the mean value |
| Age of grade III mineralization | 1 | 118 | 239,5593 | 63,36471 | 5,83319 |
|  | 3 | 124 | 234,3065 | 66,81587 | 6,00024 |

### Table 13.1: Levene Test for equality of variances and T-Test for equality of means

|  | | | | | | | | | | |
| --- | --- | --- | --- | --- | --- | --- | --- | --- | --- | --- |
|  | | Levene | | t-Test | | | | | | |
|  |  | F | Sig. | T | df | Sig. (2-sided) | Mean difference | Standard error difference | 95% confidence interval of the difference | |
|  |  |  |  |  |  |  |  |  | Lower Value | Lower Value |
| Age of Grade III mineralization | Variances are equal | ,030 | ,863 | ,627 | 240 | ,531 | 5,25287 | 8,37938 | -11,25366 | 21,75940 |
|  | Variances are not equal |  |  | ,628 | 239,997 | ,531 | 5,25287 | 8,36834 | -11,23190 | 21,73764 |

| Table 13.2: Effect sizes for independent variables | | | | | |
| --- | --- | --- | --- | --- | --- |
|  | | **Standardisierer^a^** | Point estimate | 95% confidence interval | |
|  |  |  |  | Lower bounds | Upper Bounds |
| Age mineralization Grade III | Cohen's d | 65,15627 | ,081 | -,172 | ,333 |
|  | Hedges' correction | 65,36077 | ,080 | -,171 | ,332 |
|  | Glass' Delta | 66,81587 | ,079 | -,174 | ,331 |
| a. The denominator used in the estimation of the effect sizes.  Cohen's d uses the summarized standard deviation.  Hedges' correction uses the summarized standard deviation and a correction factor.  Glass' delta uses the standard deviation of a sample from the control group. | | | | | |

### Table 13.3: Mann-Whitney U Test for sex in Grade III

|  | | | | |
| --- | --- | --- | --- | --- |
|  | Null hypothesis | Test | Sig.^a,b^ | Decision |
| 1 | The distribution of Grade III mineralization is identical for both genders | Mann-Whitney U-test for independent samples | ,298 | Maintain null hypothesis |
| a. The significance level is .050. | | | | |
| b. Asymptotic significance is indicated. | | | | |

| Table 13.4: Summary of the Mann-Whitney U-test for independent samples | |
| --- | --- |
| Total | 242 |
| Mann-Whitney-U-Test | 6749,000 |
| Wilcoxon-W | 14499,000 |
| Test statistics | 6749,000 |
| Standard error | 544,283 |
| Standardised test statistics | -1,042 |
| Asymptotic Sig. (two-sided test) | ,298 |

Both tests are not significant.

## Table 14: Kolmogorov-Smirnov-Test for normal distribution of data for Grade II mineralization

| **Kolmogorov-Smirnov-Test for a random sample** | | | |
| --- | --- | --- | --- |
|  | | | Age for grade II mineralization |
| N | | | 412 |
| Parameters of normal distribution^a,b^ | mean | | 147,6966 |
|  | standard deviation | | 31,76561 |
| Extreme differences | absolute | | ,064 |
|  | positive | | ,064 |
|  | negative | | -,037 |
| Test statistics | | | ,064 |
| Asymp. Significance^c^ (two sided) | | | ,000 |
| Monte-Carlo-Significance (two sided)^d^ | Sig. | | ,001 |
|  | 99% confidence interval | Lower limit | ,000 |
|  |  | Upper limit | ,001 |
| a. The distribution to be tested is a normal distribution | | | |
| b. Calculated from the data | | | |
| c. Significance correction according to Lilliefors. | | | |
| d. Lilliefors method based on 10000 Monte Carlo samples with a starting value of 1619197531. | | | |

## Table 15: Kolmogorov-Smirnov-Test for normal distribution of data for Grade III mineralization

| **Kolmogorov-Smirnov-Test for a random sample** | | | |
| --- | --- | --- | --- |
|  | | | Age for grade III mineralization |
| N | | | 242 |
| Parameters of normal distribution^a,b^ | mean | | 236,8678 |
|  | standard deviation | | 65,07416 |
| Extreme differences | absolute | | ,208 |
|  | positive | | ,208 |
|  | negative | | -,097 |
| Test statistics | | | ,208 |
| Asymp. Significance^c^ (two sided) | | | ,000 |
| Monte-Carlo-Significance (two sided)^d^ | Sig. | | ,000 |
|  | 99% confidence interval | Lower limit | ,000 |
|  |  | Upper limit | ,000 |
| a. The distribution to be tested is a normal distribution | | | |
| b. Calculated from the data | | | |
| c. Significance correction according to Lilliefors. | | | |
| d. Lilliefors method based on 10000 Monte Carlo samples with a starting value of 622500317. | | | |

## Table 16: Binary logistic regression analysis with age as the only predictor

*Wald Test non-significant for b (p=0.056) and const. (p=0.062)

†Model significant, however one coefficient is not significant

*Logistic regression analysis with age as predictor: Parameters from the logistic regression for grade III with age as predictor. χ² is the test statistic, p is the significance of the overall model, n is the number of individuals per breed group, exp(B) is the odds ratio giving the relative odds if age is incremented, % gives the percentage of correctly classified status of the dogs,* *and t*_0,5_ *or t*_0,9_ *is the age in days when the probability of reaching grade III is 50% or 90% respectively. b and – const. are the coefficients for the regression model, which are assessed for significance with Wald test.*

## Figure 1: Grade II mineralization in different breed groups – effect size for ANOVA

$$f=\sqrt{\frac{\eta^{2}}{1-\eta^{2}}}=\sqrt{\frac{0,088}{1-0,088}}=0,311$$

## Figure 2: Grade III mineralization in different breed groups - effect size for ANOVA

$$f=\sqrt{\frac{\eta^{2}}{1-\eta^{2}}}=\sqrt{\frac{0,071}{1-0,071}}=0,276$$

## Figure 3: Grade II chondrodystrophic dogs - effect size for T-Test

$$r=\sqrt{\frac{t^{2}}{t^{2}+df}}=\sqrt{\frac{4,355^{2}}{4,355^{2}+410}}=0,210$$

## Figure 4: Grade II chondrodystrophic dogs - effect size for effect size for Mann-Whitney U Test

$$r=\frac{Z}{\sqrt{n}}=\frac{4,050}{\sqrt{410}}=0,200$$

## Figure 5: Grade III chondrodystrophic dogs - effect size for T-Test

$$r=\sqrt{\frac{t^{2}}{t^{2}+df}}=\sqrt{\frac{1,666^{2}}{1,666^{2}+240}}=0,107$$

## Figure 6: Grade III chondrodystrophic dogs - effect size for Mann-Whitney-U-Test

$$r=\frac{-2,276}{\sqrt{242}}=0,146$$

| Figure 7: Grade II mineralization in different sexes - effect size for T-Test |
| --- |

$$r=\sqrt{\frac{t^{2}}{t^{2}+df}}=\sqrt{\frac{3,421^{2}}{3,421^{2}+410}}=0,167$$

## Figure 8: Grade II mineralization in different sexes - effect size for Mann-Whitney-U-Test

$$r=\frac{-3,623}{\sqrt{412}}=0,178$$

## Figure 9: QQ Plot of data distribution for Grade II mineralization

*Beobachteter Wert = Observed value*

*Erwarteter Wert = Expected value*


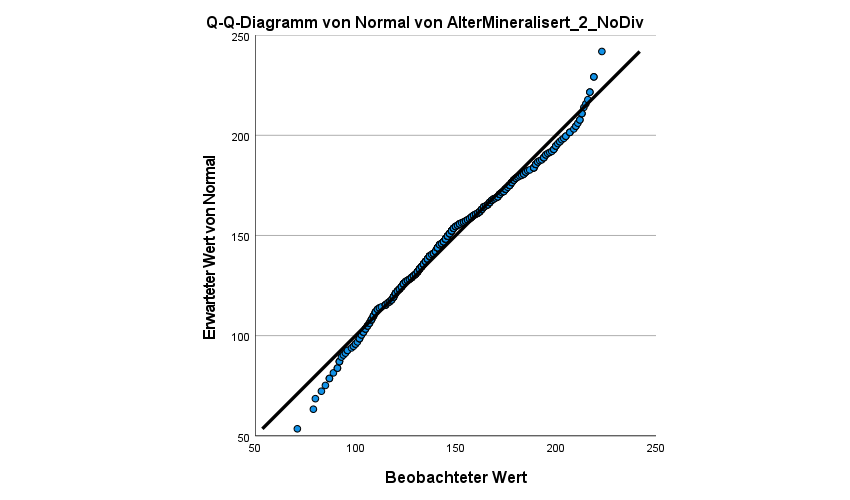


|  |
| --- |
|  |

## Figure 10: QQ Plot of data distribution for Grade III mineralization

*Beobachteter Wert = Observed value*

*Erwarteter Wert = Expected value*


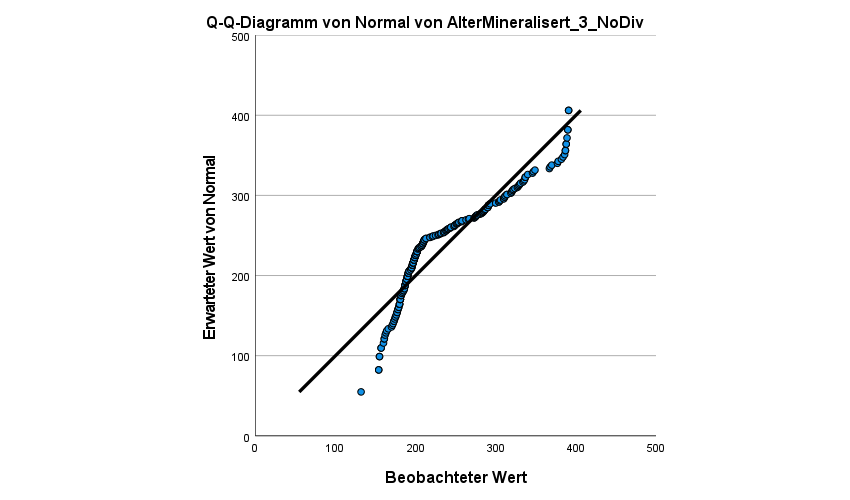

Supplement: Supplementary file 1 — vru70177‐sup‐0001‐Appendix.docx. [file VRU-67-0-s001.docx]
